# Supplementary material for: Cafeteria and Fast-Food Diets Induce Neuroinflammation, Social Deficits, but a Different Cardiometabolic Phenotype
Source: Nutrients. 2025 Nov 19;17(22):3614. doi: 10.3390/nu17223614 (PMC12655535; doi:10.3390/nu17223614)
Supplement: Supplementary file 1 [file nutrients-17-03614-s001.zip › nutrients-3941500-supplementary.pdf]

Supplementary material to the paper:

Cafeteria and Fast-food Diets Induce Neuroinflammation, Social Deficits, but a Different Cardiometabolic Phenotype

By: Andrej Feješ [1], Petronela Sušienková [1], Lucia Mihalovičová [1,2], Veronika Kunšteková [1,] Radana Gurecká [3], Veronika Borbélyová [1,] Peter Celec [1], Katarína Šebeková [1,] *

[1]Institute of Molecular Biomedicine, Faculty of Medicine, Comenius University, Bratislava; Sasinkova 4, 811 08 Bratislava, Slovakia; [2]Institute of Epidemiology and Prevention, Faculty of Public Health, Slovak Medical University, Limbová 12, 83303 Bratislava; [3] Institute of Medical Physics, Biophysics, Informatics and Telemedicine, Faculty of Medicine, Comenius University, Sasinkova 2, 811 08 Bratislava, Slovakia

Supplementary Table S1: Composition of fast-food diet (energy, macronutrients, and food additives). E numbers according to each ingredient: bun (E491, E300, E472e), pickles (E202), ketchup (glucose-fructose syrup, spice extracts), beef patty (none listed), processed cheese (E331, E332, E160a, E160c). Classification of E numbers by their purpose: *Preservative*: E202 – Potassium sorbate; *Emulsifier*: E491 – Sorbitan monostearate; E472e – Mono- and diacetyl tartaric acid esters of mono- and diglycerides of fatty acids; E331 – Sodium citrates (specifically: Trisodium citrate), E332 – Potassium citrate (specifically: Tripotassium citrate); *Antioxidant*: E330 – Ascorbic acid; *Colouring*: E160a – Carotene; E160c – Paprica extract; *Stabilizer and chelating agent to prevent discoloration*: E339 – Sodium phosphate (specifically: Disodium phosphate); *Anti-foaming agent*: E900 – Dimethylpolysiloxane.

Item	Energy (kcal/ 100g)	Fat (g/100g)	Carbohydrate (g/100g)	Protein (g/100g)	Food additives (E number)
Cheeseburger	253	11	24	13,6	E491, E300, E472e, E202, E331, E332, E160a, E160c, Glucose-fructose syrup, Spice extracts
Fries	286	14,4	32,6	4,6	Dextrose, E900, E339

Supplementary Table S2: Composition of cafeteria diet- energy, macronutrients, and food additives. Menus composition: ***Menu 1***: Item 1, Item 2, Item 3, Item 4; ***Menu 2***: Item 5, Item 6, Item 7, Item 8, Item 9; ***Menu 3***: Item 2, Item 10, Item 11, Item 12, Item 13; ***Menu 4***: Item 6, Item 14, Item 15, Item 16. **Classification of E numbers by their purpose:** *Colours*: E160a – Carotenes, E120 – Carmines, E160c – Paprica extract; *Preservatives*: E250 – Sodium nitrite, E200 – Sorbic acid, E202 – Potassium sorbate, E282 – Calcium propionate; *Antioxidants*: E300 – Ascorbic acids, E316 – Sodium erythorbate; *Sweeteners*: E420 – Sorbitol; *Emulsifiers, Stabilisers, Thickeners and Gelling Agents*: E322 – Lecithin, E481 – Sodium stearoyl-2-lactylate, E472e – Mono- and diacetyltartaric acid esters of mono- and diglycerides of fatty acids, E471 – Mono- and diglycerides of fatty acids, E414 – Gum Arabic, E492 – Sorbitan tristearate. E476 – Polyglycerol polyricinoleate, E401 – Sodium alginate, E475 – Polyglycerol esters of fatty acids, E401 – Sodium alginate; *Others*: E296 – Malic acid, E330 – Citric acid, E450 – Diphosphates, E500 – Sodium carbonates, E904 – Shellac, E451 – Triphosphates, E503 – Ammonium carbonates, E422 – Glycerol, E509 – Calcium chloride, E501 – Potassium carbonates, E524 – Sodium hydroxide, E920 - L-Cysteine, E1404-E1452 – Modified starch.

Item	Product	Energy (kcal/ 100g)	Fat (g/100g)	Carbohydrate (g/100g)	Protein (g/100g)	Fibre (g/100g)	Salt (g/100g)	Food additives (E number)

1	Donut	404	24	8.2	6,1	2,1	1	E481, E472e, E471, E322, E450, E500, E160a, E300, E904, E7414
2	Salami	496	44	2.1	23	0	2,5	E451, E450, E316, E120, E250
3	Chocolate cream	574	37	21.2	6,4	0	0,09	E322
4	Salted crackers	452	17	4.3	9,4	2,9	2,3	E503, E500, E322
5	Muffins	452	22,9	25.2	5,1	1,6	0,9	E420, E422, E450, E500, E200
6	Gouda cheese	352	28	0.1	25	0	1,3	E509
7	Cookies	514	27	2.3	4,5	1,6	0,89	E503, E500, E450, E322, E160a
8	Cocoa cookies with vanilla flavour filling	472	19	7.3	5,6	2,9	0,76	E503, E501, E500, E322, E524
9	Salted potato chips	542	35	18.2	5,6	4,4	0,95	-
10	Brownie	468	27	19.2	5	1,7	0,38	E492, E322, E476, E202, E330, E401, E500, E450
11	Soft caramels	382	5,2	18.3	5,4	0	0,15	-
12	Marshmallow	324	< 0,5	16.3	4,3	<0,5	0,18	-
13	Salted sticks	382	3,8	12.3	13	4	4	E524, E503
14	Croissant	455	28	43	5,5	1,9	0,57	E471, E475, E282, E202, E401, E300, E920
15	Chocolate bar with caramel filling	493	24	5.3	4,6	0	0,39	E322, E500
16	Tortilla chips - Nacho cheese flavour	475	24,3	25.2	5,5	0	1,9	E330, E296, E160c. E1404-E1452

Supplementary Table S3: Additional physiological variables. CAF- cafeteria diet, FFD- fast-food diet, RBC- red blood cell count, HCT- hematocrit, LDL-C- low-density lipoprotein cholesterol, NGAL- neutrophil gelatinase-

associated lipocalin, ncDNA- nuclear fraction of extracellular DNA, mtDNA- mitochondrial fraction of extracellular DNA. *- CAF vs. CTRL; #- FFD vs. CTRL; $- CAF vs. FFD; #- $p<0.05$; **/$$- $p<0.01$.

Parameter	CTRL	FFD	CAF	F	p value
RBC (1012/L)	8.8±0.4	9.2±0.3 #	8.9±0.3	3.7	<0.05
HCT (%)	51±2.3	50.3±2.5	49.1±2	1.9	0.176
Diuresis (mL/ 24 hours)	28.9±8.1	22±4.8	16.1±6.6 *	8.8	<0.001
Urine osmolality (mOsmol)	439.1±164.6	393.4±97.6	773.2±323.7 **/ $$	8.9	<0.001
Plasma Creatinine (mg/mL)	36.3±2.3	34.7±3.1	36.4±3.1	1.2	0.325
Creatinine Clearance (ml/min/ g kidney)	7.4±1.1	6.9±2.7	5.8±2.8	1.2	0.330
Proteinuria (mg/ 24 hours)	0.06±0.1	0.05±0.1	0.1±0.1	2.5	0.103
NGAL (ng/ 24 hours)	102.5±193.8	153.2±179.2	356.5±397.1	2.3	0.118
Plasma LDL-C (mmol/mL)	0.5±0.1	0.5±0.2	0.7±0.4	3.0	0.068
Plasma Albumin (mmol/L)	1.3±0.2	1.7±0.6	1.7±0.4	2.6	0.095
ncDNA (GE/mL)	3.8±0.4	3.8±0.6	3.5±0.4	0.8	0.456
mtDNA (GE/mL)	1.5×106±1.1×106	4.2×106±8.6×106	2.5×106±2×106	0.7	0.518
